# No strong associations between temperature and the host–parasite interaction in wild stickleback

**DOI:** 10.1111/jfb.15107

**Published:** 2022-07-15

**Authors:** Hanna M. V. Granroth‐Wilding, Ulrika Candolin

**Affiliations:** ^1^ Organismal and Evolutionary Biology Research Programme, Faculty of Biological and Environmental Sciences University of Helsinki Helsinki Finland

**Keywords:** cestode, climate change, ecological parasitology, *Gasterosteus aculeatus*, host–parasite interaction, *Schistocephalus solidus*

## Abstract

As climate change progresses, thermal stress is expected to alter the way that host organisms respond to infections by pathogens and parasites, with consequences for the fitness and therefore population processes of both host and parasite. The authors used a correlational natural experiment to examine how temperature differences shape the impact of the cestode parasite *Schistocephalus solidus* on its host, the three‐spined stickleback (*Gasterosteus aculeatus*). Previous laboratory work has found that high temperatures benefit *S. solidus* while being detrimental to the stickleback. The present study sought to emulate this design in the wild, repeatedly sampling naturally infected and uninfected fish at matched warmer and cooler locations in the Baltic Sea.

In this wild study, the authors found little evidence that temperature was associated with the host–parasite interaction. Although infection reduced host condition and reproductive status overall, these effects did not vary with temperature. Host fitness indicators correlated to some extent with temperature, with cooler capture sites associated with larger size but warmer sites with improved reproductive potential. Parasite fitness (prevalence or size) was not correlated with temperature at the capture site.

These mismatches between laboratory and field outcomes illustrate how findings from well‐controlled laboratory experiments may not fully reflect processes in more variable natural settings. Nonetheless, the findings of this study indicate that temperature can influence host fitness regardless of infection, with potential consequences for both host demography and parasite transmission dynamics in this complex system.

## 1 INTRODUCTION

Parasites and pathogens are a key influence on individual fitness and population processes in wild hosts (Altizer *et al*., 2013; Hudson *et al*., 2002; Tompkins *et al*., 2011). Infection has been shown to alter host traits of eco‐evolutionary importance from mortality and fecundity to mate choice and migration strategy (Hamilton & Zuk, 1982; Hoye *et al*., 2016; Hudson *et al*., 2002; Watson, 2013). Nonetheless, wild hosts simultaneously face many other stresses and resource demands that must be traded off against investment in defending against and coping with infection (Albery *et al*., 2020; Sheldon & Verhulst, 1996; Stearns, 1992). Environmental factors can influence both hosts' resource balance and parasite prevalence, and therefore how hosts are impacted by infection (Granroth‐Wilding *et al*., 2014; Shearer & Ezenwa, 2020). Such effects are anticipated to be magnified by anthropogenic environmental change, which has the potential to bring novel physiological stress and behavioural changes (Bairlein, 2016; Candolin & Wong, 2012; Seehausen *et al*., 1997), with implications for both hosts and parasites (Altizer *et al*., 2013; Budria & Candolin, 2015). This is particularly important in the context of stressed natural populations when infection impacts on individual vital rates, such as reproductive success and mortality, that together determine host population growth rates (Agnew & Koella, 1999; Albon *et al*., 2002; Ferreira *et al*., 2019; Pelletier & Garant, 2012; Smith *et al*., 2008; Valenzuela‐Sanchez *et al*., 2021; Watson, 2013).

Temperature is a well‐documented environmental variable predicted to change substantially over the coming decades as a consequence of human activity (Masson‐Delmotte *et al*., 2021). Thermal change has therefore been a particular focus of research into climate‐driven changes in the occurrence and outcome of parasitism (Altizer *et al*., 2013; Barber *et al*., 2016; Harvell *et al*., 2002, 2007; Lafferty, 2009). Thermally stressed hosts may be less able to defend against parasite infection (*e.g.*, Bradley *et al*., 2019) and, conversely, infected individuals may be more susceptible to thermal stress (Greenspan *et al*., 2017). In chipmunks, temperature is positively correlated with infection by parasitic botflies (Paquette *et al*., 2020), and in corals increased temperatures are associated with increased severity of outbreaks of black band disease (Harvell *et al*., 2007). Therefore, warmer temperatures have been linked with range shifts and increased virulence of parasites in humans (Bartlow *et al*., 2019; Caminade *et al*., 2019) as well as among wild hosts (Kent *et al*., 2020; Turner *et al*., 2021). Nonetheless, the complexity of any host–parasite‐environment system means that the links between temperature, parasite prevalence and impact on host populations often remain unclear.

Environmental conditions can influence the biology of the parasite as well as the host (Agnew & Koella, 1999; Paull *et al*., 2012). The resulting changes to the host–parasite interaction could have complex and counterintuitive consequences for host fitness (Barber *et al*., 2016). For example, in a frog‐trematode system, laboratory experiments showed that although parasites increased their output in warmer temperatures, they also developed faster; the resulting temporal mismatch between infective stages and receptive hosts ultimately decreased the population‐level impact of the parasite (Paull *et al*., 2012). Conversely, in three‐spined stickleback, Macnab and Barber (2012) showed that higher temperatures have multiplicative negative effect on host success: in warm water, development of the fish host is impaired, whereas the development of its cestode parasite is favoured. This effect is further exaggerated as the parasite manipulates the host's behaviour such that the fish seeks out warmer water.

Despite similar observations from several wild systems that environmental stress can interact with parasitism to affect the performance or fitness of individual hosts, fundamental differences between the systems make it difficult to identify general patterns (Altizer *et al*., 2013; Valenzuela‐Sanchez *et al*., 2021). Therefore, the extent to which complex effects shown in laboratory studies translate into meaningful patterns in the wild remains poorly understood. An alternative approach to testing the importance of thermally moderated changes to host–parasite interactions in the wild is to examine host–parasite interactions in thermally divergent ecosystems. Controlled laboratory manipulations of infections and/or temperature and detailed observations of their outcomes give clear predictions for expected patterns of host responses in the wild. Mismatches between laboratory and field results then point towards areas where our understanding requires further development. Such potential mismatches are of particular interest where infection has non‐lethal impacts on fitness‐related traits (Watson, 2013): a fecundity reduction observed in captivity may, for example, have little impact on population processes in the wild if host mortality mainly occurs before reproduction. Nonetheless, this approach can be applied only in study systems amenable to both laboratory manipulation and detailed field surveys.

The three‐spined stickleback *Gasterosteus aculeatus* (Linnaeus 1758), a small fish common across the temperate Northern Hemisphere, and its cestode parasite *Schistocephalus solidus* constitute a well‐established laboratory and wild system for host–parasite interactions in which infection reduces host fitness in terms of both survival and reproductive output (Barber, 2013; Barber & Scharsack, 2010; Budria & Candolin, 2015). Natural temperature gradients and anthropogenic environmental change are associated with *S. solidus* prevalence in the wild (Budria & Candolin, 2015; Karvonen *et al*., 2013), and higher temperatures have been shown in the laboratory to be simultaneously beneficial to the parasite and detrimental to the fish (Franke *et al*., 2017; Macnab & Barber, 2012; Scharsack *et al*., 2021). This previous work also provides clear predictions for the quantitative relationships the authors expect to find between parasitism, temperature and host performance in the wild.

The present study examines how the prevalence and impact on hosts of *S. solidus solidus* infection varies with temperature and host phenotype in natural populations of stickleback, testing whether increasing thermal stress increases parasites' impact on the host in the wild as it does in a laboratory setting (Macnab & Barber, 2012). To mirror experimental temperature manipulation, the authors use a comparative approach, sampling populations throughout the breeding season at six sites with different thermal regimes. By selecting pairs of closely located sites at each location, the authors capture natural variability while controlling for confounding factors such as microclimate in different parts of the archipelago. They tested whether, in this natural experiment, higher temperatures negatively impacted host fitness while promoting parasite fitness, and examined whether infection altered hosts' preference to favour warmer water.

## 2 MATERIALS AND METHODS

**FIGURE 1:**
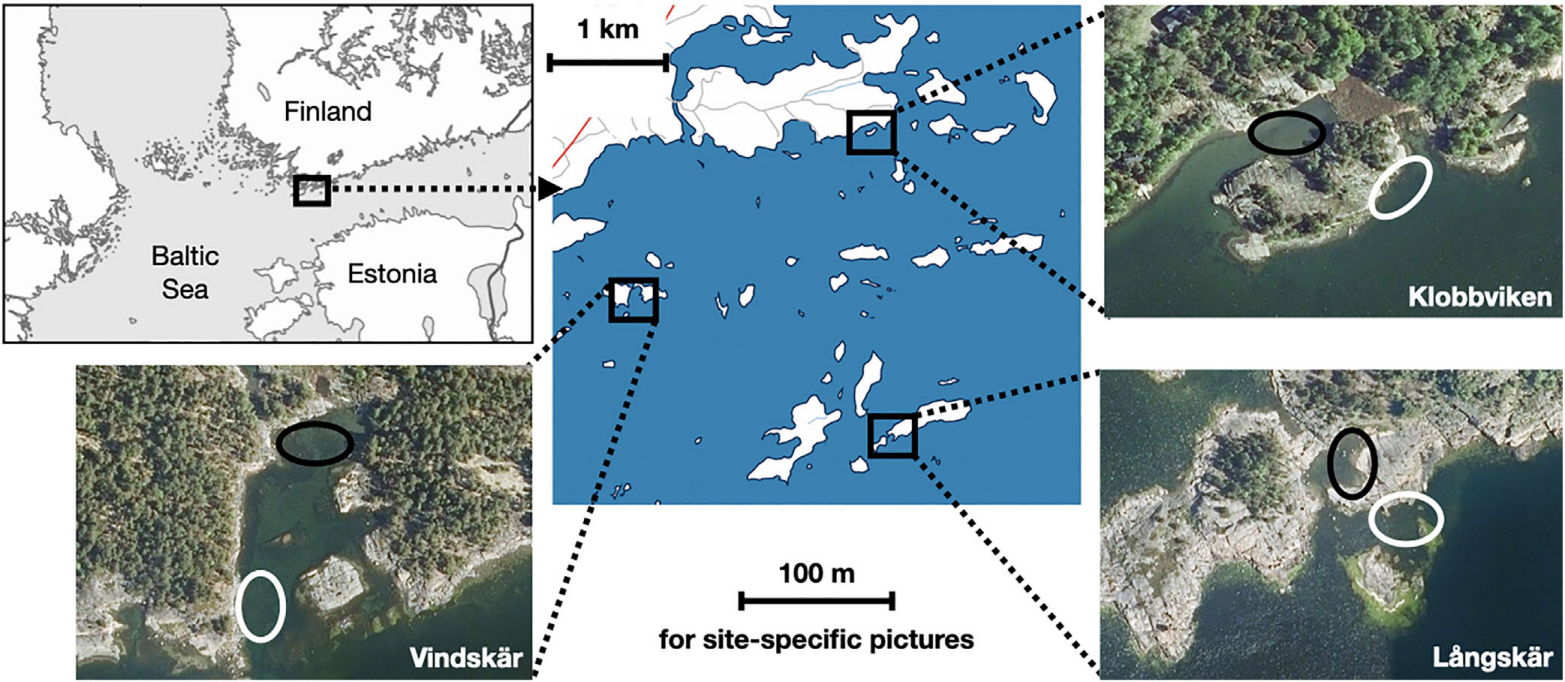
The location in Finland (top left map) of the field site (central map) and within that, sampling sites (aerial photographs, with site names). Black squares show areas enlarged in other maps; ellipses show specific sampling areas, with inner locations in black and outer locations in white

Field sampling was carried out at the Hangö Peninsula in southern Finland (Figure 1) in June–July of 2016 and 2018. Wild stickleback populations in the Baltic Sea are already experiencing warmer and less predictable temperature regimes (Mikkonen *et al*., 2015). Sticklebacks in this population are understood to winter in cool, deep water, and return in May to warmer coastal waters to breed. Once breeding is complete, the 2+ cohort dies, whereas younger cohorts migrate back to open water in the autumn.

Stickleback populations were sampled throughout the summer once breeding populations were established. In 2016, sampling was conducted at six sites, a matched pair at each of three locations (Figure 1). Each location was a bay in the Southern Finnish archipelago, situated at a range of distances out to sea and therefore cooler water: on the mainland (Klobbviken), in the middle archipelago (Vindskär) and on the seaward edge of the archipelago (Långskär). At each bay, two sites were sampled: inside the bay, a sheltered setting that would experience warmer temperatures, and on the outer edge of the bay, a setting more exposed to the open sea with more constant and cooler temperatures. This design gave three matched pairs of sampling sites (warm, variable inner bay and cool, constant outer bay) on a decreasing temperature gradient away from the mainland. Thermometers deployed at each site at a depth of *c*. 40 cm recorded the temperature every 2 h for the duration of the study. Data were collected from the thermometers twice during the study; one was not retrievable at the end of the study (Långskär outer, the most exposed site), such that temperature data were obtained for June for all six sites but only for five across the whole study period.

Sampling was carried out five times at each location at an interval of 7–16 days, as weather permitted, from peak breeding in early June and to the end of July, when few adult stickleback remain in shallow waters. Both sites at each location were sampled on the same day. Fish were captured using transparent plexiglass minnow traps with wings to direct the fish towards the opening (Candolin & Voigt, 2001). Six traps were used at each site, placed close to vegetation at a depth range of 30–100 cm over a distance of 20–30 m along the shoreline. Traps were deployed for *c*. 14 h overnight, from 19.00–20.30 hours to 09.30–11.00 hours to avoid mortality in the traps during the hotter part of the day. Fish from all six traps at each site were combined, immediately taken back to the laboratory and held in flow‐through containers for up to 36 h until further examination.

From each sampling site, up to 50 fish (all fish if the total catch was fewer than 50) were weighed, measured for length and killed by decapitation. Lethal sampling was required to obtain accurate data on each individual's infection status and reproductive status, as well as data on the number and size of parasites. All institutional ethical guidelines were met. Each fish was then examined for visible external parasites and dissected to be examined for visible internal parasites and confirm its sex. All *S. solidus* individuals were removed from the host's body cavity and weighed. Multiple infections were found in only two fish across the whole study, which both hosted two *S. solidus* plerocercoids; in these cases the authors used total parasite mass in the analyses. Stickleback reproductive status was scored on a four‐point scale, for females based on the extent of egg development visible on dissection (no yolk on any eggs through to all eggs well yolked, *i.e.*, ready to spawn) and for males based on external nuptial coloration (no coloration through to deep red throat and bright blue eyes). When more than 50 fish were caught, the entire catch was transferred to a large holding tank and 50 individuals were haphazardly netted out after agitating the water to ensure all fish were moving around the water, with every other net set aside for release rather than killing so as to decrease bias towards less mobile individuals. This approach did not affect prevalence of *S. solidus* (*χ* ^2^ = 0.172, *P* = 0.679). The remaining fish were not killed but were counted and released back into the wild.

In May–July 2018, supporting data were collected in parallel with a separate long‐term monitoring study. Here, only inner sites were sampled, using three traps to catch up to 30 fish for dissection. Thermometers were deployed at these sites for two representative weeks in peak season (19 June–4 July), reflecting the availability of temperature data in 2016.

### 2.1 Analysis

Temperature effects were examined in terms of mean June temperature, calculated for each site as for the period 8–26 June in 2016, from when the first trap was deployed at the last location to the beginning of a sudden inflow of cold water that markedly altered the temperature profile of all sites, and for the period 19 June–4 July in 2018. Inner sites tended to be slightly warmer and more variable than outer sites (Table 1; Supporting Information Figure S1), and temperature means and variances were correlated across sites (Spearman's *r* ^2^ = 0.77, *t* = 2.40, *P* = 0.074); therefore, the authors focus on mean temperature in this study.

**TABLE 1:** Summary of sample sizes, thermal profiles and host and parasite traits for stickleback and their cestode parasite *S. solidus* at each of six sampling sites in 2016, an inner and outer site at each of three bays on the Southern Finnish coast, and three sampling sites in 2018, only the inner bays

Site	Location in archipelago	Mean June temp. (°C)	June temp. variance (°C)	Total nr. fish captured	Sex ratio (# males/# females)	Parasite prevalence (# infected/# uninfected)	Mean length ± s.e. (mm)
*2016*							
Klobbviken inner	Mainland	14.9	3.3	822	0.51 (98/95)	0.07 (13/180)	51.4 ± 0.3
Klobbviken outer	Mainland	13.5	1.1	234	0.35 (45/82)	0.07 (9/118)	51.3 ± 0.3
Vindskär inner	Middle archipelago	14.8	2.3	324	0.61 (89/56)	0.05 (7/138)	51.2 ± 0.3
Vindskär outer	Middle archipelago	13.1	1.4	195	0.46 (37/43)	0.11 (9/71)	52.2 ± 0.4
Långskär inner	Open water edge	15.9	6.2	342	0.53 (80/70)	0.03 (4/146)	51.4 ± 0.3
Långskär outer	Open water edge	12.0	2.0	80	0.31 (24/54)	0.02 (1/77)	53.1 ± 0.4
*2018*							
Klobbviken inner	Mainland	14.9	6.8	204	0.50 (101/103)	0.05 (10/194)	49.7 ± 0.5
Vindskär inner	Middle archipelago	12.8	3.9	35	0.66 (23/11)	0.09 (3/32)	52.4 ± 0.8
Långskär inner	Open water edge	14.8	12.7	49	0.49 (24/25)	0.16 (8/41)	53.6 ± 0.7

*Note*: Sex ratio, parasite prevalence and length were measured only on dissected fish (max. 50 per site per sampling occasion), and sample sizes are smaller than the total population captured.

First, the authors tested for associations between temperature and traits in both hosts and parasites, as well as the host–parasite relationship, using data pooled across both years. An initial inspection showed no clear temporal patterns in fish or parasite populations across sampling sites within or between the years (see Supporting Information). The following response variables were tested: for hosts, size (length), condition (weight/length^3^, where weight did not include the parasite in infected individuals and was adjusted to reproductive status in females, based on a linear regression, to account for egg mass) and reproductive status; for parasites, prevalence (whether a host was infected or not) and plerocercoid weight; and to illustrate the host–parasite interaction, plerocercoid weight relative to host weight. The impact of infection was tested using infection status as a predictor of host traits. Main effects of temperature (an extrinsic influence on the host–parasite interaction), between‐host variation (an intrinsic influence) and infection itself were tested simultaneously in a full model that was simplified by step‐wise removal of effects with the least significant effect. To examine whether temperature influenced how parasites impacted the host, interactions between host phenotype and temperature or infection, or between infection and temperature, were tested as a single interaction term in separate models.

To compare more directly to previous laboratory results (Macnab & Barber, 2012), the authors also examined whether temperature affected how host growth, parasite growth and infection impact changed with time, through the season. This was tested using, respectively, a temperature * date interaction to predict host length and parasite weight, and an infection * temperature * date interaction to predict host condition (weight/length^3^).

Lastly, the authors examined whether infected fish within each bay's population showed a preference for the warmer water at the inner site, as expected from laboratory results (Macnab & Barber, 2012). They tested this on the 2016 data only using site type (inner/outer) as the response variable and infection status (infected or not) as the predictor.

All analysis used (generalized) linear mixed models [(G)LMMs] with location fitted as a random factor, to account for potential similarities within the populations at each site, and year fitted as a two‐level fixed factor. All models of host traits included sex as a predictor, to account for sexual dimorphism. Continuous responses used Gaussian errors and an identity link, and binary responses (sex, infection status) used binomial errors and a logit link. Reproductive status was scaled to a range from 0 to 1 and also tested in binomial models. To ensure model convergence, predictors were scaled where necessary by an appropriate constant. Model selection proceeded backwards, removing the least significant term one by one until all remaining terms were significant, starting from a maximal model for each response trait as shown in Table 2.

**TABLE 2:** Summaries of the minimal models testing the influence of temperature (mean or variance) on host phenotype, temperature interacting with host phenotype on parasite fitness, and temperature interacting with host phenotype and infection with Schistocephalus on host fitness

		Minimal model summary					
Response	Maximal model(s) predictors	Predictor	Effect size	Std. error	Test statistic	Test statistic value	*P*
*Host phenotype*							
Sex	Mean temp.	(Intercept)	−4.17	0.94	*z*	−4.44	0.000
		Mean June temp.	0.29	0.06	*z*	4.44	0.000
	Temp. variance	(Intercept)	−0.76	0.31	*z*	−2.44	0.015
		Temp. variance	0.23	0.06	*z*	3.91	0.000
Length	Mean temp. + sex	(Intercept)	55.8	1.6	*t*	35.35	0.000
		Mean June temp.	−0.2	0.1	*t*	−1.80	0.072
		Sex	−2.7	0.3	*t*	−10.26	0.000
	Temp. variance + sex	(Intercept)	53.0	0.3	*t*	204.38	0.000
		Sex	−2.7	0.3	*t*	−10.68	0.000
*Parasite fitness*							
Prevalence	Mean temp. * host length + mean temp. * host sex	(Intercept)	−13.59	2.32	*z*	−5.86	0.000
	Temp. variance * host length + temp. variance * host sex	Length	2.02	0.42	*z*	4.75	0.000
Weight	Mean temp. * host length + mean temp. * host sex	(Intercept)	−0.016	0.195	*t*	−0.08	0.936
	Temp. variance * host length + temp. variance * host sex	Length	0.007	0.004	*t*	1.98	0.055
*Host fitness indicators (including parasite impacts)*							
Condition	Mean temp. * host length * infection status + mean temp. * host sex Mean temp. * host length + mean temp. * host sex * infection status	(Intercept)	0.012	0.001	*t*	9.211	0.000
		Length	0.000	0.000	*t*	−4.831	0.000
		Mean June temp.	0.000	0.000	*t*	5.832	0.000
		Infected	−0.002	0.000	*t*	−6.949	0.000
		Sex	0.004	0.001	*t*	2.789	0.005
		Mean June temp. : Sex	0.000	0.000	*t*	−3.227	0.001
	Temp. variance * host length * infection status + temp. variance * host sex Temp. variance * host length + temp. variance * host sex * infection status	(Intercept)	0.017	0.001	*t*	18.277	0.000
		Length	0.000	0.000	*t*	−4.741	0.000
		Temp. variance	0.000	0.000	*t*	5.245	0.000
		Infected	−0.002	0.000	*t*	−6.980	0.000
		Sex	−0.001	0.000	*t*	−4.947	0.000
Condition (males only)	Mean temp. * host length * infection status	(Intercept)	0.018	0.001	*t*	16.132	0.000
		Length	0.000	0.000	*t*	−4.878	0.000
		Infected	−0.001	0.000	*t*	−3.539	0.001
Reprod. status	Mean temp. * host length * infection status + mean temp. * host sex Mean temp. * host length + mean temp. * host sex * infection status	(Intercept)	−2.5	0.6	*t*	−4.53	0.000
		Mean June temp.	0.4	0.0	*t*	9.55	0.000
		Infected	−0.7	0.2	*t*	−4.52	0.000
		Sex	4.7	0.9	*t*	5.43	0.000
		Mean June temp. : Sex	−0.4	0.1	*t*	−6.05	0.000
	Temp. variance * host length * infection status + temp. variance * host sex Temp. variance * host length + temp. variance * host sex * infection status	(Intercept)	2.0	0.2	*t*	8.33	0.000
		Temp. variance	0.3	0.0	*t*	7.86	0.000
		Infected	−0.7	0.2	*t*	−4.44	0.000
		Sex	0.0	0.1	*t*	0.08	0.934
		Temp. variance : Sex	−0.2	0.0	*t*	−4.36	0.000
Reprod. status (males)	Mean temp. * host length * infection status + mean temp. * host condition	(Intercept)	2.0	0.7	*t*	2.88	0.004
		Mean June temp.	0.0	0.0	*t*	0.33	0.743
		Infected	5.6	3.1	*t*	1.79	0.075
		Mean June temp. : Infected	−0.4	0.2	*t*	−1.94	0.053

*Note*: For several responses, different maximal models (including different interactions) yielded the same minimal model. In some cases (see main text), interactions were found to be significant that were driven by only one site. In these cases, the minimal models shown here are those robust to the exclusion of those sites. All “Sex” terms are shown for males compared to females, and "Infected" terms for infected compared to uninfected hosts.

## 3 RESULTS

### 3.1 Role of temperature in parasite and host fitness

The sampling sites showed different thermal profiles, with mean temperatures ranging from 12.0 to 15.9°C and temperature variance ranging from 1.1 to 6.2°C and similar temperatures in both study years (Table 1; Supporting Information Figure S1). Although temperature mean and variance were strongly correlated across sites, mean temperature was consistently more strongly associated with both host and parasite traits than was temperature variance (Table 2; all minimal models using temperature variance ΔAIC > 4 compared to the same model using mean temperature instead).

**FIGURE 2:**
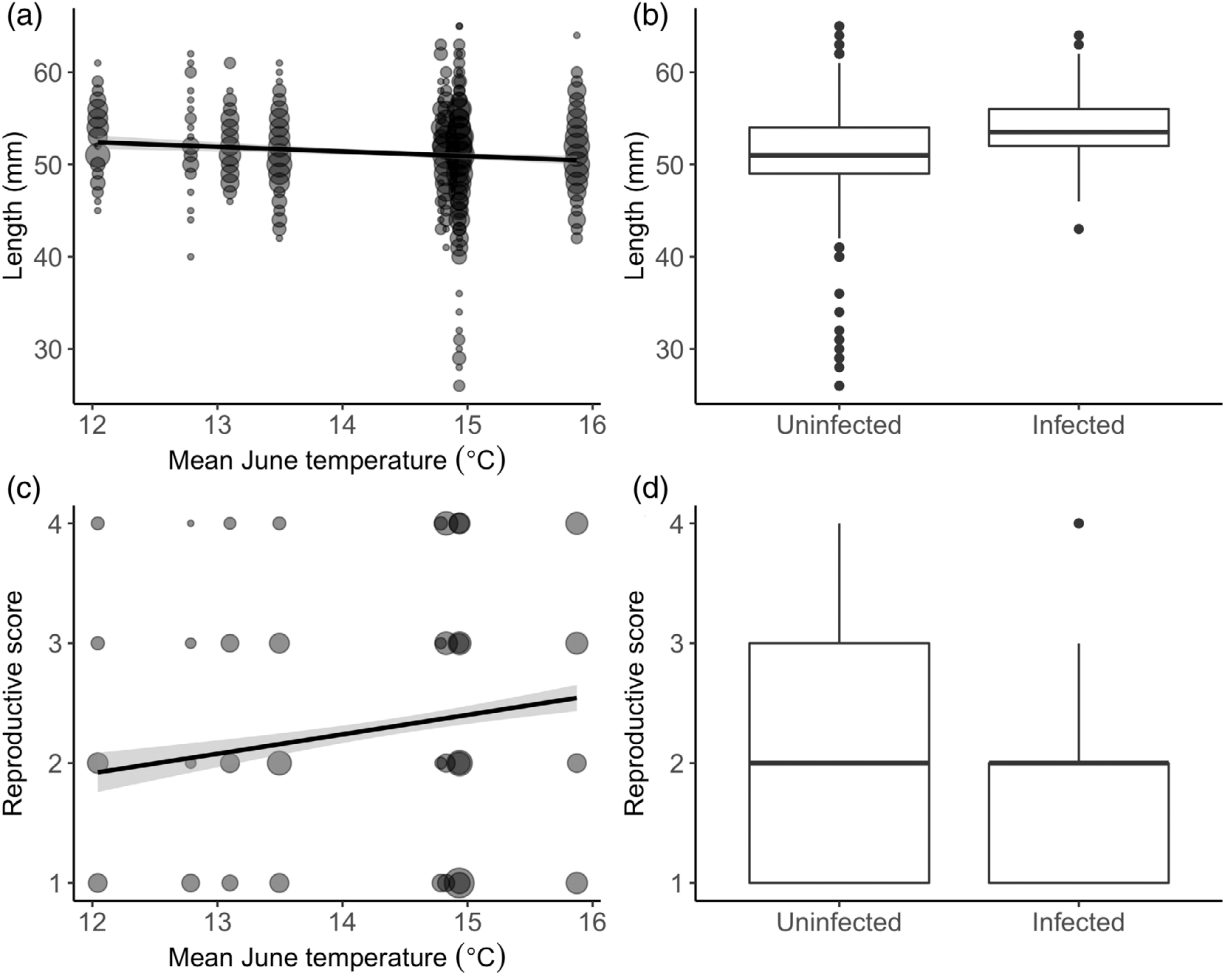
Associations of temperature (left panels a and c) and *S. solidus* infection (right panels b and d) with host phenotype (length, top panels a and b) and host fitness as indicated by reproductive status (bottom panels c and d). The points show raw data, with larger points indicating a greater number of individuals, and the fitted lines show predictions with shaded 95% c.i., derived from the minimal models given in Table 2 (fitted without random effects). For clarity, the length model does not include the sex effect; males were overall shorter than females (Table 2)

The authors found no link between temperature and *S. solidus* fitness indicators, either prevalence and size (Table 2). Instead, parasite prevalence was associated with between‐host differences, with infection more common among larger hosts (Table 2; Figure 2). Nonetheless, parasite size did not vary with host size. Temperature was consistently not associated with parasite fitness irrespective of host size (effect of host length * temperature interaction: on parasite prevalence, scaled effect size 12.2 ± 16.2, *z* = 0.754, *P* = 0.451; on parasite size, −0.00 ± 0.01, *t* = −0.43, *P* = 0.672). There was no indication that parasite prevalence or growth rate varied with temperature over the sampling period, or that these parasite fitness indicators changed through time overall (effect of date on both parasite prevalence and size, either in interaction with temperature or as main effect in models in Table 1, all *P* > 0.09).

Although temperature was not linked with parasite fitness, it was associated with host fitness: among uninfected fish, individuals from warmer water had higher reproductive scores, although their condition (*i.e.*, weight in relation to length) did not vary with temperature (Table 2; Figure 2). Moreover, in warmer locations, fish of both sexes were smaller (Table 2; Figure 2). Fish size increased slightly over the season, irrespective of temperature (effect of date on length: 0.03 ± 0.01 mm/day, *t* = 2.84, *P* = 0.005; date * temperature interaction, 0.01 ± 0.01, *t* = 1.12, *P* = 0.263).

### 3.2 Impact of infection on hosts

Overall, *S. solidus* infection was associated with lower host condition and lower host reproductive score (effect of infection, as main effect in addition to models in Table 1: on fish condition, −0.002 ± 0.000, *t* = −6.81, *P* < 0.001; on fish reproductive status, −1.68 ± 0.37, *z* = −4.52, *P* < 0.001; Figure 2). The impact of infection on hosts was consistent across temperatures and host sizes, despite the link between temperature and host fitness and between host phenotype and parasite fitness (infection * temperature interaction and infection * host length interaction on either host condition or reproductive score, *P* > 0.18). There was no evidence that infection affected host growth or reproductive development differently over the course of the season (effect of date * infection on host length, 0.04 ± 0.03, *t* = 1.30, *P* = 0.193; effect of date * infection on host reproductive score, −1.5 ± 1.1, *z* = −1.43, *P* = 0.152), nor that any association of temperature with the impact of infection on host condition changed over the course of the season (temperature * date * infection interaction on host condition, 7.72 × 10^−6^ ± 1.65 × 10^−5^, *t* = 0.467, *P* = 0.640). Parasite weight relative to host weight, an indicator of the host–parasite interaction, also did not show any link with temperature (in addition to host length, temperature effect −0.00 ± 0.01, *t* = −0.32, *P* = 0.7496). Nonetheless, in a pattern not detected in absolute parasite weight, parasites were lighter relative to the host in larger (longer) hosts (−0.01 ± 0.00, *t* = −2.81, *P* = 0.007).

There was no evidence that infected fish preferred the warmer, inner site at each location (number of fish in inner *vs*. outer predicted by infection status in binomial GLMM, effect size 0.31 ± 0.32, *z* = 0.97, *P* = 0.332).

## 4 DISCUSSION

In this study the authors have used a correlational, natural experimental approach to investigate whether temperature is associated with the outcome of infection for both a wild stickleback host and its cestode parasite. Previous laboratory work found that high temperatures benefitted the parasite while being detrimental to the host (Macnab & Barber, 2012; Scharsack *et al*., 2021). Nonetheless, in wild, free‐ranging fish, the authors found little evidence that the outcome of the host–parasite interaction was linked with temperature. In this study, thermal environment was associated with host fitness but not parasite fitness. Moreover, although infection reduced host body condition and reproductive status overall, these effects did not vary with temperature.

In laboratory experiments on young stickleback hosts, Macnab and Barber (2012) found that the infecting *S. solidus* grew faster in warmer water, whereas on the contrary, cooler water benefitted host fitness. Accordingly, in behavioural tests, infected hosts actively preferred warmer water than uninfected hosts originating from a similar temperature. This study sought to mirror this design in the wild. Although the results partly reflected the favourability of lower temperatures to hosts, which were larger in cooler water, the authors also found that warmer water favoured other fitness indicators in the wild fish, notably reproductive status. In addition, the sampling found no evidence that infected wild fish preferred warmer water inside the bay more than did uninfected fish. This suggests that cooler temperatures may benefit growth, as measured in the experiments on young fish (Macnab & Barber, 2012; Scharsack *et al*., 2021), but not reproductive potential in adults, which were the focus of the present study where all fish appeared sexually mature. Taken together, these different results indicate that temperature could have pleiotropic effects on fitness across the stickleback's lifetime. The results for adult fish do not allow the authors to differentiate whether warmer water causally drove maturation, or whether mature fish moved to warmer water to nest and mate. Irrespective of the mechanism, given that reproduction is an essential fitness component, it would be fruitful in further work to determine whether warmer water is also detrimental to the growth of young stickleback in the wild, and if so, which life stages are more sensitive to temperature.

The authors found no strong evidence of an association in the wild between temperature and either parasites' fitness (prevalence or size) or the host–parasite interaction (impact of infection on host traits). This is perhaps unexpected, given that studies in many other wild systems have identified an effect of temperature on parasite prevalence (*e.g.*, Paquette *et al*., 2020) or infection intensity (*e.g.*, Harvell *et al*., 2007). The scope of this study, covering only 2 years and a temperature scale within natural limits in this relatively short period, gave the authors only limited power to pick out temperature associations from the myriad other factors influencing parasite fitness in this setting. Instead, they found an association between infection and host phenotype, with parasites found more frequently in larger hosts (mirroring Barber, 2005), which in turn were found more frequently in cooler water. Despite this chain of links, the authors did not capture a direct connection between temperature and parasite fitness. Accordingly, although infection was linked with host condition and reproductive status overall, these fitness indicators increased similarly with temperature in infected and uninfected fish. It is worth noting that this temperature insensitivity of *S. solidus* prevalence in the wild is consistent with infection success in the laboratory being similar across temperatures (Macnab & Barber, 2012).

Nonetheless, these various lab‐field mismatches highlight the need in disease ecology to constantly question whether responses in well‐controlled laboratory experiments, or indeed in theoretical models, are meaningful to more variable and complex natural settings. Notably, although the correlational nature of this study prevents us from drawing conclusions regarding causality in the observed relationships, the results suggest a smaller impact of temperature on host–parasite interactions in a wild population than in an equivalent lab experiment. This mismatch is in the opposite direction to what is often assumed or demonstrated, namely that laboratory populations, freed from resource constraints and associated investment trade‐offs, should experience less marked parasite impacts than wild populations (Candolin & Voigt, 2001). On the contrary, a wild setting brings in variation in a huge number of other unmeasured factors that are excluded from a laboratory experiment, whose influence on the host–parasite interaction could obscure any weaker influence of temperature. The authors used free‐living hosts with the intent of capturing as much as possible of real host–parasite interactions playing out in the wild, but this approach is at the same time limited by our lack of knowledge around the history of infection and thermal experience of the host individuals. At least some infections are likely to have been established some time before the start of this study period, perhaps even before the winter (Confer *et al*., 2012), weakening the link between the measured temperature and parasite fitness indicators. At the same time, given the low prevalence found, naïve fish were likely also exposed to infective parasites throughout the sampling period. The fact that the authors did not observe an increase in parasite size through time indicates that hosts with large parasites disappearing from the population were balanced by new infections, with smaller parasites, being established at a similar rate. If infections were established substantially before this study began, then the prevalences observed could also be affected by temperatures at the time of initial exposure, which can affect the success of *S. solidus* establishment (Scharsack *et al*., 2021). Moreover, infection outcomes depend on the hosts’ temperature of origin, such that hosts are less tolerant of infection at unfamiliar temperatures (Franke *et al*., 2017). Nonetheless, in the present study, thermal experience since an initial infection may only play a limited role as it is likely to be broadly similar across all hosts, because they spend the non‐breeding season in deeper, cooler water, whereas differences in temperature are most marked during the summer study period. The stickleback‐*S. solidus* system is ideally suited to further investigations of the role of infection history: natural thermal profiles for the non‐breeding period could be replicated in the laboratory and controlled parasite exposures conducted, with these fish then compared to free‐living hosts using exclosures during the breeding season. Such approaches would also allow for control of other confounding factors in the natural setting.

In addition to unknowns around hosts' thermal history, the sampling sites of this study are likely to have varied in many other environmental factors beyond the temperature that was measured. Differences in predation pressure, for example, could have important consequences: infection with *S. solidus* changes sticklebacks' behaviour such that they move towards the surface and are less mobile, which facilitates predation by seabirds, the parasite's final host (Barber, 2013). Based on pilot observations, the authors found that seabird predation was infrequent at all sites. This meant that it was not practicable to measure, but also likely to have only a limited role in shaping between‐site variation in prevalence, although we cannot rule out unobserved predation by fish playing a similar role. The sampling sites may also have differed in aspects such as food availability or quality, or the prevalence of infective intermediate copepod hosts of *S. solidus*, which was beyond the scope of this study to quantify.

Working in a wild setting also shaped the way in which the authors’ key focus, temperature, varied within their data set. This study covered a range of temperatures that was similar in breadth to previous laboratory studies, yet it remained within the natural range for Baltic shoreline environments. This may have been insufficient to capture the patterns in host–parasite relationships seen in more extreme thermal conditions, such as the increased parasite prevalence observed in sticklebacks in volcanically heated waters (Karvonen *et al*., 2013). Moreover, temperatures beyond the current natural range may be particularly informative to predicting how climate change will affect host–parasite dynamics, as the thermal environment changes beyond currently normal fluctuations, including more frequent extreme weather events (Masson‐Delmotte *et al*., 2021; Ummenhofer & Meehl, 2017). Indeed, recent work in the stickleback‐*S. solidus* system indicates that warming may impede host defences against infection only above a threshold temperature (Scharsack *et al*., 2021), which this natural experiment may not have captured. Nonetheless, this study diverged from this picture in that mean temperature explained more variation in host traits than did temperature variance.

In addition to temperature, the authors found that host phenotype was associated with parasite prevalence and thermal regime, with larger individuals having a higher infection prevalence and being found preferentially in cooler water. That fish were larger in cooler locations could indicate either differences in growth rate *in situ*, greater selective mortality (likely through predation by larger fish) of small individuals in cooler water, or bigger individuals moving to cooler areas. Unfortunately, the correlative, population‐level data of this study do not allow the authors to differentiate between these possibilities. Similarly, infections being more frequent in larger hosts may be due to the growing cestode causing earlier mortality of smaller infected individuals (Lynsdale *et al*., 2017; Sol *et al*., 2003), removing them preferentially from the population. On the contrary, the authors’ finding that larger hosts harboured relatively smaller parasites suggests that larger hosts are more resistant to the infection, supporting the idea that small infected fish disappear earlier from the population. They were not able to dig into such mechanisms using their observational data, but this is a fruitful avenue for further work: between‐host differences are known to be important in shaping the prevalence or outcome of infection in many host–parasite systems (Granroth‐Wilding *et al*., 2014; Lynsdale *et al*., 2020; Paquette *et al*., 2020), and understanding and accounting for such differences are essential to realistically model the demographic role of parasites in natural populations (Watson, 2013; Wilber *et al*., 2016), particularly in a changing environment (Altizer *et al*., 2013; Ezenwa & Jolles, 2015). Indeed, the results suggest potential for *Schistocehpalus* infection to influence stickleback population processes over a longer period, given its marked suppression of fertility in females (55% of uninfected but only 20% of infected females were close to or already reproductive) and negative impact on host condition. This study system would be flexible for more controlled experiments in a natural setting, such as using exclosures to control fish movement between habitats or predation pressure, which would begin to unravel the causality behind the patterns that have been demonstrated here.

In summary, the authors found that *S. solidus* infection in wild Baltic sticklebacks was associated with poorer host fitness indicators, that infection was associated with host size such that larger hosts more often harboured parasites, and that larger hosts were associated with cooler water. Despite these links, thermal regime did not appear to be an important determinant of population‐level patterns in parasite prevalence or the impact of infection on host performance. These findings contrast markedly with previous laboratory work in this system that showed warmer water to simultaneously benefit the parasite and negatively impact the host (Macnab & Barber, 2012). This study thus highlights the care that must be taken when extrapolating laboratory findings to natural settings. Nonetheless, the findings of this study indicate the potential for temperature changes to interact with natural host–parasite relationships to influence host fitness and therefore demography as well as parasite fitness and transmission dynamics (Altizer *et al*., 2013; Ezenwa & Jolles, 2015; Valenzuela‐Sanchez *et al*., 2021).

## AUTHOR CONTRIBUTIONS

H.M.V.G.‐W. conceived the idea of the study, obtained funding, conducted field and lab work, conducted the statistical analyses and wrote the manuscript. U.C. developed the idea, supported the practical and statistical work, and wrote the manuscript.

## Supporting information


**FIGURE S1** Temporal trends in water temperature at six sampling sites in the Tvärminne Archipelago, southern Finland. At each of the three locations (Klobbviken, Vindskär and Långskär, progressively further out to the open sea), two sites were sampled, one inside and the other on the outer edge of a bay. The light grey line shows the raw time series, the thick black line a GAM with four knots fitted to each location, the translucent white field around the smooth shows its standard errors and the grey point and error bars at the left of each panel show the site's mean temperature and temperature variance in June (the values used in the analysis).
**FIGURE S2** Temporal trends at the six sampling sites in (A) host population size, (B) parasite prevalence in terms of whether each host was infected, with points jittered around 0 and 1 for clarity, (C) host length (a key aspect of between‐individual variation) and (D) host condition (a fitness indicator). Host population sizes (panel A) were measured five times at each site over the course of the study; the graph shows a smoothed trend for clarity. In panels B–D, points show the measured data and lines the fitted values from a minimal model of the interacting influence of time and location. Locations are denoted by the colour of the points/lines; inner sites are shown with solid points and lines and outer sites with hollow points and dotted lines. There was no difference between sites in parasite prevalence, so the fitted line in panel B is not colour‐coded.
