# Supplementary material for: No strong associations between temperature and the host–parasite interaction in wild stickleback
Source: J Fish Biol. 2022 Jul 15;101(3):453–63. doi: 10.1111/jfb.15107 (PMC9545309; doi:10.1111/jfb.15107)

**SUPPLEMENTARY INFORMATION**

This supplementary material outlines difference between sampling sites over the course of the sampling season, in terms of temperature profile (Fig. S1) and host and parasite characteristics (Fig. S2).

Stickleback populations showed a humped, long-tailed distribution through time whose shape differed between sites (Fig. S2). Peak population sizes were largest at inner sites and the occurred latest at the most seaward location (Långskär), but with no visually obvious link with temperature regime (Fig. S1). Among the captured fish, sex ratio became more male-biased and body condition decreased through the season, while the largest (longest) fish were captured in the middle of the study (table 2, Fig. S2). All of these trends differed between sampling sites (table 2, Fig. S2), but again with no visible link to temperature. In contrast to the variation in host populations and traits, neither parasite prevalence nor size showed any temporal trends or differences between sites (tables 1 and 2, Fig. S2).

**Figure S1**. Temporal trends in water temperature at six sampling sites in the Tvärminne Archipelago, southern Finland. At each of three locations (Klobbviken, Vindskär & Långskär, progressively further out to the open sea), two sites were sampled, one inside and the other on the outer edge of a bay. The light grey line shows the raw time series, the thick black line a GAM with 4 knots fitted to each location, the translucent white field around the smooth shows its standard errors, and the grey point and error bars at the left of each panel shows the site’s mean temperature and temperature variance in June (the values used in the analysis).


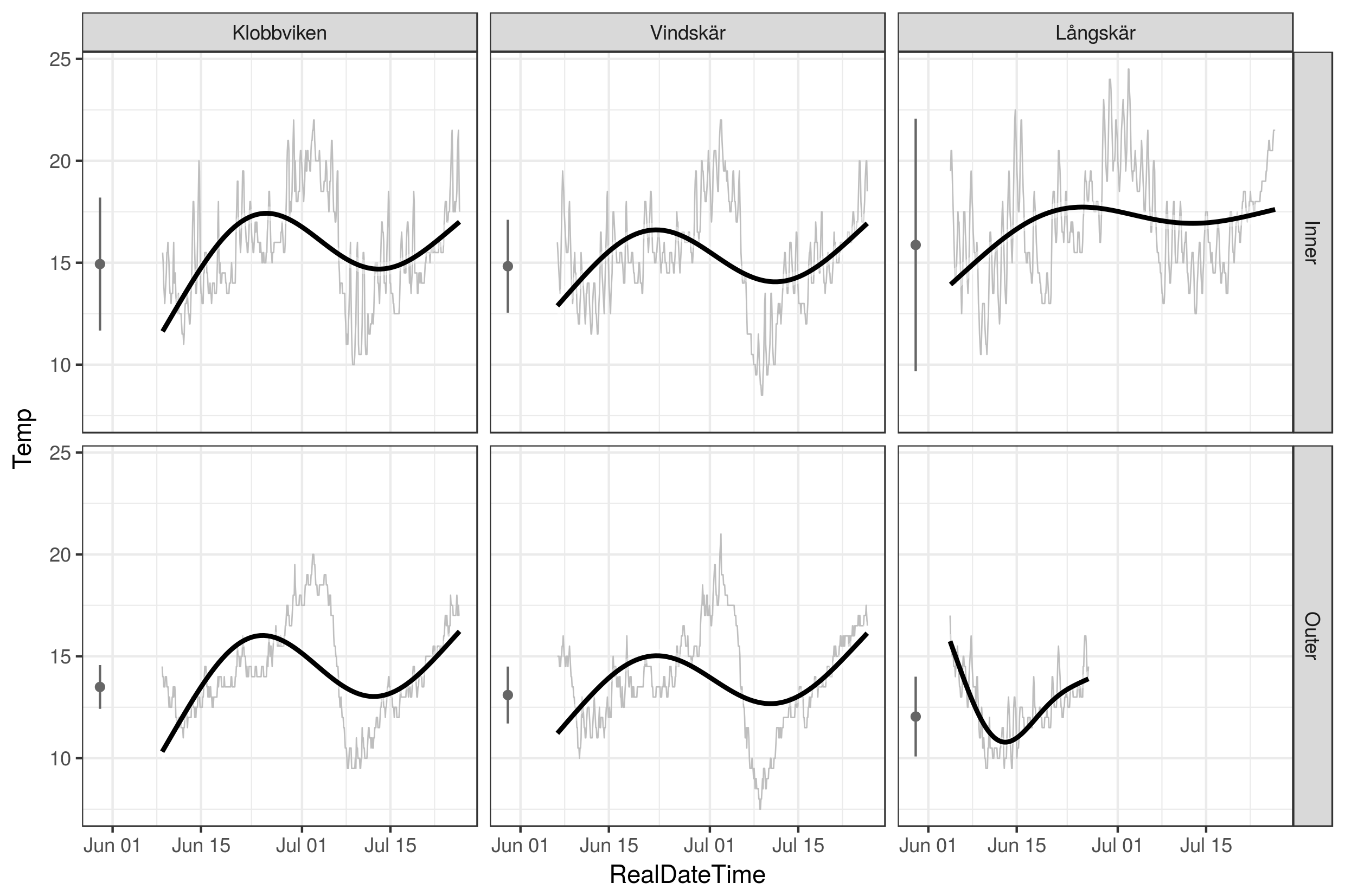


**Figure S2.** Temporal trends at the six sampling sites in: (A) host population size, (B) parasite prevalence in terms of whether each host was infected, with points jittered around 0 and 1 for clarity, (C) host length (a key aspect of between-individual variation) and (D) host condition (a fitness indicator). Host population sizes (panel A) were measured 5 times at each site over the course of the study; the graph shows a smoothed trend for clarity. In panels B-D, points show the measured data and lines the fitted values from a minimal model of the interacting influence of time and location (see table X). Locations are denoted by the colour of the points/lines; inner sites are shown with solid points and lines and outer sites with hollow points and dotted lines. There was no difference between sites in parasite prevalence, so the fitted line in panel B is not colour-coded.


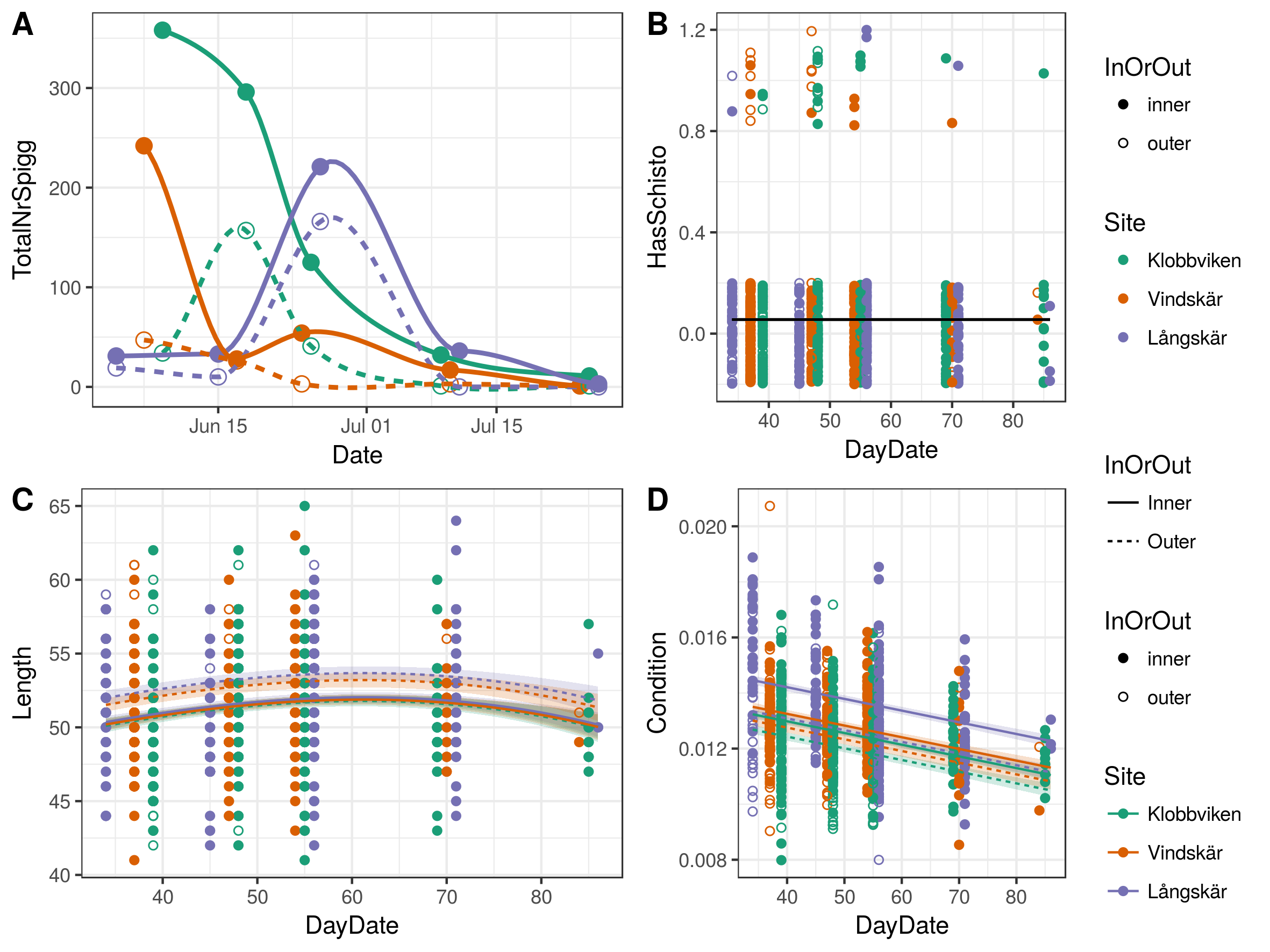

Supplement: Supplementary file 1 — FIGURE S1 Temporal trends in water temperature at six sampling sites in the Tvärminne Archipelago, southern Finland. At each of the three locations (Klobbviken, Vindskär and Långskär, progressively further out to the open sea), two sites were sampled, one inside and the other on the outer edge of a bay. The light grey line shows the raw time series, the thick black line a GAM with four knots fitted to each location, the translucent white field around the smooth shows its standard errors and the grey point and error bars at the left of each panel show the site's mean temperature and temperature variance in June (the values used in the analysis). FIGURE S2 Temporal trends at the six sampling sites in (A) host population size, (B) parasite prevalence in terms of whether each host was infected, with points jittered around 0 and 1 for clarity, (C) host length (a key aspect of between‐individual variation) and (D) host condition (a fitness indicator). Host population sizes (panel A) were measured five times at each site over the course of the study; the graph shows a smoothed trend for clarity. In panels B–D, points show the measured data and lines the fitted values from a minimal model of the interacting influence of time and location. Locations are denoted by the colour of the points/lines; inner sites are shown with solid points and lines and outer sites with hollow points and dotted lines. There was no difference between sites in parasite prevalence, so the fitted line in panel B is not colour‐coded. [file JFB-101-453-s001.docx]
